# A Significant Inhibitory Effect on Advanced Glycation End Product Formation by Catechin as the Major Metabolite of Lotus Seedpod Oligomeric Procyanidins

**DOI:** 10.3390/nu6083230

**Published:** 2014-08-13

**Authors:** Qian Wu, Shuyi Li, Xiaopeng Li, Xiaoyan Fu, Yong Sui, Tingting Guo, Bijun Xie, Zhida Sun

**Affiliations:** 1 Natural Product Laboratory, Department of Food Science and Technology, Huazhong Agricultural University, Wuhan, Hubei 430070, China; E-Mails: qianwu@umass.edu (Q.W.); 15392909677@189.cn (X.L.); suiyong0429@126.com (Y.S.); gtt693532@126.com (T.G.); bijunxie@sina.com (B.X.); 2 College of food science and engineering, Wuhan Polytechnic University, Wuhan, Hubei 430070, China; E-Mail: lishuyisz@sina.com; 3 College of Food and Biology Science and Technology, Chutian College, Huazhong Agricultural University, Wuhan, Hubei 430070, China; E-Mail: fuxiaoyan001@163.com

**Keywords:** advanced glycation end products (AGEs), lotus seedpod oligomeric procyanidins (LSOPC), metabolites, carbonyl, antioxidant

## Abstract

Several lines of evidence suggested that B-type procyanidin oligomers from lotus seedpod (LSOPC) may effectively modulate the formation of advanced glycation end products (AGEs). *In vivo*, LSOPC is metabolized by intestinal flora to become various kinds of phenolic compounds that possess potent antioxidant activities. However, few reports of the absorption and metabolism of LSOPC have been revealed. In the present study, rats were orally administered with LSOPC at a dose of 300 mg/kg body weight. The metabolites of LSOPC in urine were elucidated by HPLC-MS/MS analysis 24 h post-administration. Eight major metabolites were significantly increased by the administration of 300 mg/kg of LSOPC (*p* < 0.01). The anti-glycative activity of LSOPC and its metabolites were investigated. The results showed that LSOPC and catechin had greater anti-glycative activities than other metabolites, which were positively correlated to their carbonyl scavenging activities and antioxidant capacities.

## 1. Introduction

Advanced glycation of proteins had been initially investigated by food and nutrition biochemists [1]. The Maillard reaction, a nonenzymatic process, is initiated when amino groups on proteins are exposed to reducing sugars, lipids or nucleic acids. It generates the first reversible Schiff base adducts and subsequently more stable Amadori rearrangement products. First, the carbonyl group from a reducing sugar and an unprotonated amine group from a protein produce a nucleophilic addition reaction to form a freely reversible Schiff base. This is subsequently stabilized after rearrangement into Amadori products or Heyns products according to the type of sugar involved (aldoses or ketoses). Through a series of oxidative and nonoxidative reactions, early glycated products are formed [2]. Further reactions (cross-linkages and polymerization) lead to the formation of advanced glycation end products (AGEs) [3]. AGEs were originally characterized by a yellow-brown fluorescent color and by an ability to form cross-links with and between amino groups, but the term is now used for a broad range of advanced products during the glycation process (also called the “Maillard reaction”).

Considering the multifactorial pathways and complexity of reactions involved in AGE formation, AGE inhibitors may be a strategy to reduce the occurrence of AGE-associated diseases. Since oxidative processes are implicated in both the formation and toxicity of AGEs, dietary antioxidants can play an important role in mitigating both processes. Repeatedly identified as effective antioxidants and AGE inhibitors [4], plant phenolics are of particular interest. The wealth of food and medicinal plants used in aboriginal and popular cultures worldwide offer a unique opportunity to provide management options that are pharmacologically, culturally and economically relevant to diverse at-risk populations.

Lotus seedpod is a part of lotus, which is rich in B-type procyanidins. The mean degree of polymerization of LSOPC was 3.21 with 74.2% catechin and 25.8% epicatechin in the terminal units and 26.0%, 43.1% and 30.9% of catechin, epicatechin and epigallocatechin in the extensive units, respectively. Our laboratory has established the proper extraction technology of LSOPC [5]. Furthermore, previous studies showed various physiological and biological functions of LSOPC in terms of antioxidant activities [6]. Some researchers indicated that LSOPC played a potential role in the treatment of cognitive impairment caused by Alzheimer’s disease [7]. Recently, our previous studies have showed that LSOPC could effectively inhibit the formation of AGEs under simulated physiological environment [8]. Scalbert *et al*. [9] reported that the concentration of intact polyphenols in plasma rarely exceeds 1 μmol/L, and their urinary recovery ranges from 1 to 25% of the ingested dose. Phenolic acids, like caffeic acid, are easily absorbed through the gut barrier, whereas large molecular weight polyphenols, such as procyanidins, are very poorly absorbed [10,11]. Furthermore, flavonoid intake has been estimated to be as high as 1 g/day, and the plasma levels of such flavonoids and their metabolites are markedly increased after consumption, which may offer some protection against glucose-induced protein damage. It should be mentioned that recent studies with flavonoids, such as rutin and quercetin, have demonstrated intracellular uptake, although the mechanisms involved remain unknown [12,13]. However, the studies on the metabolites and metabolism pathways of LSOPC *in vivo* are rarely reported. 

In this paper, urine metabolite analysis was used with the aim of providing insight into the absorption and metabolism of LSOPC following the oral intake of 300 mg/kg body weight by rats. Using HPLC-MS/MS, the major metabolites of LSOPC were identified. Furthermore, the inhibitory effect of LSOPC and its metabolites were compared on AGE formation. Then, the proposed structure-function relationship of LSOPC and its metabolites were classified on anti-glycation effects, carbonyl scavenging capacities and antioxidant activities. The biological activities of metabolites of LSOPC contribute to exploring the anti-AGE mechanisms of LSOPC.

## 2. Materials and Methods

### 2.1. Ethics Statement

All experimental procedures involving animals followed the Guiding Principles in the Care and Use of Animals and were approved by the ethics committee of the Reference Laboratory for the test of Veterinary Drug Residues of Huazhong Agricultural University (SYXK 2007-0044), Hubei Province, China. We made all efforts to minimize suffering, and the animals were killed by cervical dislocation under anesthesia.

### 2.2. Materials

Mature lotus seedpods of *Nelumbo nucifera* Gaertn. (cultivar: Number 2 Wuhan plant) were obtained from Honghu Lantian (Hubei, China) in late July, 2012, and identified by Professor Xueming Ni from the Department of Botany, Wuhan Plant Institute of the Chinese Academy of Science. (+)-Catechin, ferulic acid, caffeic acid, syringic acid, *m*-coumaric acid, 3-hydroxybenzoic acid, 3-hydroxyphenylacetic acid and 3-hydroxyphenylpropionic acid were purchased from Sigma Chemical Co. (St. Louis, MO, USA). The β-glucuronidase demonstrated both β-glucuronidase (600 U/mg solid) and sulfatase (50 U/mg solid) activity. Methanol and formic acid (HPLC grade) were purchased from Fisher Scientific (Massachusetts, USA). Phosphate buffer saline (PBS, pH 7.4), bovine serum albumin (BSA) and d-glucose were purchased from Sinopharm Chemical Reagent (Shanghai, China). All other chemicals were of analytical grade.

### 2.3. Preparation of LSOPC

Fresh lotus seedpod fragments were extracted using 70% ethanol at 60 °C for 1.5 h. The crude procyanidin aqueous solution was loaded onto an AB-8 resin (weak polarity macroporous resin, 0.3–1.25 mm particle size, Nankai Hecheng Science & Technology Co., Tianjin, China) column (15 × 3.5 cm, ID), and the fraction eluted by 70% ethanol was collected. The eluent was evaporated, and the procyanidin extract of lotus seedpod was obtained. Subsequently, they were extracted by ethyl acetate to get the oligomeric procyanidins of lotus seedpod (LSOPC), which included catechin monomers, B-type procyanidins dimers, trimers and a few tetramers by LC-MS analysis [14].

### 2.4. Butanol-HCl Assay

The procyanidin content of LSOPC was measured using the butanol-HCl method [15]. Briefly, LSOPC was dissolved in methanol at 0.1 mg/mL. To a 10-mL screw cap tube, 6 mL of the acid butanol reagent (950 mL of butanol with 50 mL concentrated HCl), 1.0 aliquot of LSOPC and 0.2 mL of the iron reagent (2% ferric ammonium sulfate in 2 M HCl) were added and vortexed. The tube was capped loosely and placed in a boiling water bath for 45 min, then cooled for 15 min. The final result was detected at 546 nm by a UV-2100 spectrophotometer (Unico Instrument Co. Ltd., Shanghai, China).

### 2.5. Experimental Animals and Diets

Sprague-Dawley male rats (*n* = 10) were obtained at 6 weeks from Animal Committee of Tongji Medical College (Wuhan, China). They were kept in a controlled environment at 23 °C and 55% relative humidity under a 12 h dark-light cycle, with free access to a pelleted diet (Tongji Medical College, Wuhan, China; comprising 24.0% protein, 3.5% lipids and 60.5% carbohydrate) and deionized water for 1 week. 

### 2.6. Analysis of B-type Procyanidins and Their Metabolites in Urine

Sprague-Dawley male rats (*n* = 10) weighing 210 ± 15 g were randomly divided into two groups (*n* = 5). Prior to administration of LSOPC, rats were fasted for 12 h, but had access to deionized water. The LSOPC was dissolved in 30 mg/mL physiological saline and administered orally to rats at dose of 300 mg/kg body weight. The physiological saline was administered orally to the other group as a control. All of the animals were placed in metabolic cages, one rat per cage (Jiayuan Technology Co. Ltd., Beijing, China). All urine samples excreted from 0 to 24 h post-administration were collected from the bottom of the metabolic cage under chilled conditions using an ice bath and stored at −80 °C before analysis, referring to Gonthier’s method [16].

Urine samples (~10 mL) containing B-type procyanidins and their metabolites were acidified to pH 5.5 with 0.6 mol/L acetic acid and incubated at 37 °C for 60 min in the presence of 10 KU β-glucuronidase with sulfatase activity. The sample was then centrifuged at 3000× *g* at 4 °C for 10 min, and the supernatant was removed. After further acidification to pH 2 with 6 mol/L HCl, the urine was extracted with ethyl acetate × 3. The ethyl acetate extracts were collected and reduced to dryness. The extract was dissolved in 1.5 mL of methanol containing 0.1% HCl for LC-MS analysis. 

A Symmetry C18 column (4.6 × 250 mm, 5 μm, waters, Ireland) was used on an SHIMADZU liquid chromatography 106 with a diode array detector (Shimadzu Co., Kyoto, Japan), and the mobile phases were (A) 0.2% v/v aqueous acetic acid and (B) acetonitrile. Elution conditions were as follows: a linear gradient from 5% to 15% B in 10 min, from 15% to 20% B in 5 min, from 20% to 40% B in 20 min, from 40% to 50% B in 10 min and from 50% to 5% B in 5 min, at a flow rate of 1.0 mL/min. The absorbance of the fluent was monitored at 280 nm using a diode array detector (DAD); meanwhile, the eluent was also detected by mass spectrometer. The mass fragmentation experiments were performed on an electrospray ionization (ESI) mass spectrometer with a negative ion mode. Fragmentor voltage, 100 V; capillary voltage, 2500 V; nebulizing pressure, 30 psi; dry gas temperature, 300 °C; and mass range, *m*/*z* 100–2200 [14]. Phenolic acids metabolites in rat urine were identified by retention times and molecular weights to their standard substances, respectively.

### 2.7. Inhibition of AGE Formation

The procedure was based on previous methods [17] with minor modifications. The incubation mixtures were in a final volume of 1.0 mL, containing bovine serum albumin (BSA, 5.0 mg/mL) and d-glucose (36 mg/mL) and vehicle (F_control_) or the inhibitor (F_sample_), LSOPC, catechin at 0.01, 0.02, 0.03, 0.04, 0.05, 0.1, 0.5 mg/mL concentrations and metabolites at 0.1, 0.5, 1, 1.5, 2, 2.5 mg/mL. All of these solutions were dissolved in 0.2 M phosphate buffer saline (pH 7.4) containing 3 mM sodium azide. The mixtures in screw-capped test tubes were incubated in quintuplicate in a constant temperature water bath at 37 °C for 35 days.

Following incubation, all treatments were diluted to 4 mL to quantitatively assess the formation of fluorescent AGEs using a spectrofluorometer (Shimadzu RF-5301) at excitation and emission wavelengths of 370 nm and 440 nm, respectively. F_blank_ (BSA and vehicle) was subtracted from all results and the percent inhibition then calculated as:

%inhibition = [1 − (F_sample_)/(F_control_)] × 100 (1)

IC_50_ values were calculated from %inhibition values obtained at all tested concentrations.

### 2.8. Methylglyoxal Scavenging

Methylglyoxal scavenging was tested by using a published method with additional modifications [18]. Firstly, MGO (5 mM), PD (derivatization agent, 20 mM), DQ (internal standard, 5 mM), inhibitor (5 mM) were freshly prepared in phosphate buffer saline (PBS, 50 mM, pH 7.4); the inhibitor (LSOPC and metabolites) was dispersed in phosphate buffer saline to 2.5 mg/mL. Zero-point-five milliliters of the prepared MGO solution were mixed with 0.5 mL of PBS (blank) and inhibitor. After mixing, the mixtures were incubated in a water bath at 37 °C. Samples were taken out after one hour, then cooled for 5 min. Subsequently, 0.25 mL of derivatization agent (20 mM PD) and 0.25 mL of internal standard (5 mM DQ) were added and stirred vigorously for 5 s. The tubes were kept at 37 °C for 30 min for the derivatization reaction between MGO and PD to complete. HPLC analysis of incubation media was performed on an SHIMADZU liquid chromatograph 106 with a diode array detector (Shimadzu Co., Kyoto, Japan). Compound separation was carried out in a Symmetry C18 column (4.6 × 250 mm, 5 μm, waters, Ireland). Mobile phases were composed of 0.1% formic acid in deionized water (mobile Phase A) and pure methanol (mobile Phase B). The flow rate was 1 mL/min, and the injection volume was 15 μL. The linear gradient for elution was: 0–3 min, 5%–50% B; 3–16 min, 50%–50% B; 16–17 min, 50%–90% B; 17–19 min, 90%–90% B; 19–20 min, 90%–5% B; followed by 5 min of re-equilibration of the column. The total running time was 25 min, and chromatograms were recorded at 315 nm. The amounts of unreacted MGO in the samples could be worked out on the basis of the ratios of the peak areas of MQ and DQ. The percentage decrease in MGO can be calculated using the following equation:

MGO decrease percentage = [(amounts of MGO in control − amounts of MGO in sample)/amounts of MGO in control] × 100%
(2)

### 2.9. 1,1-Diphenyl-2-picrylhydrazyl (DPPH) Radical Scavenging Activity of LSOPC and Its Metabolites

The scavenging effect of the samples on DPPH radical were evaluated according to the method of Yang *et al*. [19] with some modifications. Different extracts at various concentrations ranging from 4.0 to 100.0 µg were dissolved in 2.0 mL of deionized water. Then sample solution was mixed with 2.0 mL of freshly prepared 0.15 mM DPPH ethanolic solution. After incubation in the dark at 37 °C for 30 min, the absorbance of each solution was determined at 517 nm. The negative control was without any extracts. The scavenging effect of the sample was measured by monitoring the decrease in absorbance at 517 nm in comparison with the negative control.

### 2.10. Total Antioxidant Capability (T-AOC) of LSOPC and Its Metabolites

The total antioxidant capability (T-AOC) of LSOPC, catechin and metabolites was estimated by the total antioxidant capacity assay kit (A015, 100T, Nanjing Jiancheng Bioengineering Institute, Nanjing, China). The sample concentration was 0.05 mg/mL, and the T-AOC was expressed as mL/mg.

### 2.11. Statistical Analyses and Graph Drawing

The data of the samples were analyzed by SPSS 18.0 (expressed as the mean ± SD). IC_50_ was calculated by probit regression with SPSS. The graph was drawn by OriginPro 8.0.

## 3. Results and Discussion

### 3.1. Analysis of Procyanidin Content in the Oligomeric Procyanidins of Lotus Seedpod (LSOPC)

Using the butanol-HCl assay, we found that LSOPC contained abundant procyanidins. The content of procyanidin of LSOPC was 106.22% ± 0.46% compared with grape seed procyanidins, which was used as a standard product. The content of procyanidins in grape seed is 95%.

### 3.2. Determination of Metabolites in Rat Urine

**Figure 1:**
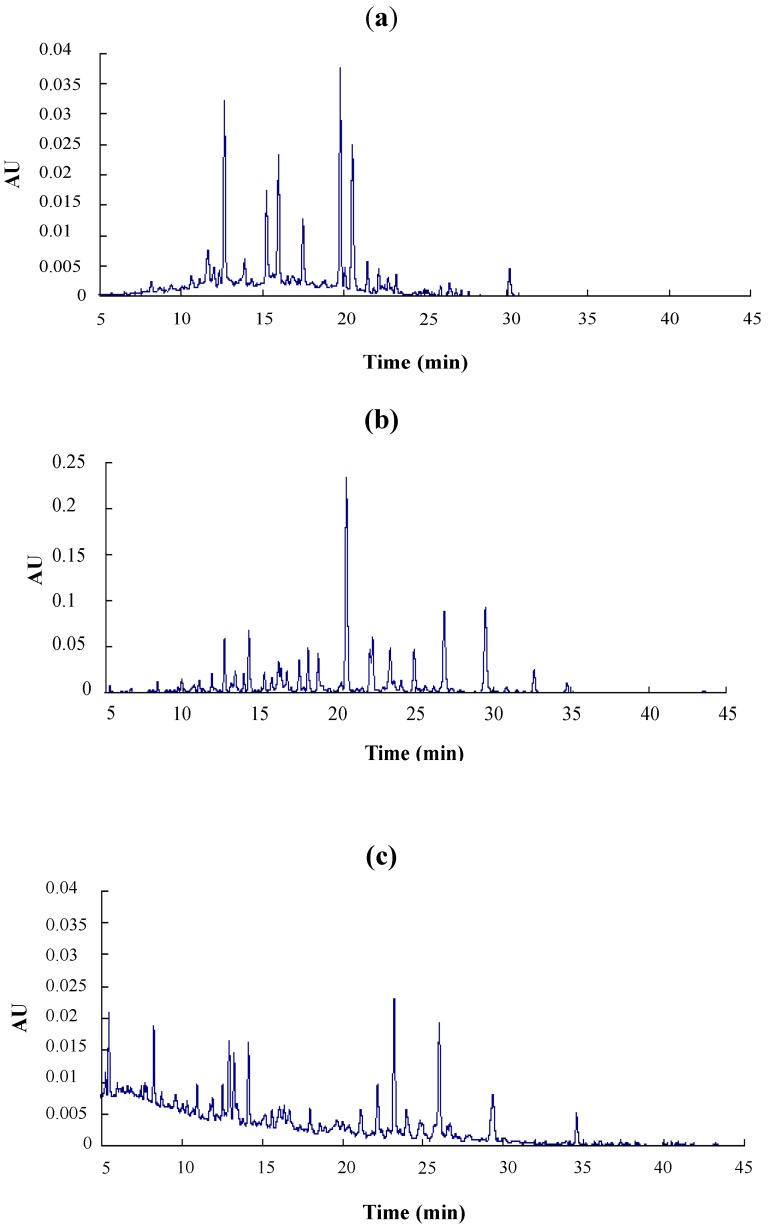
RP-HPLC chromatogram of LSOPC (**a**) (0.6 mg/mL) and their urinary metabolites in rats; (**b**) of the experimental group with an LSOPC administration dose of 300 mg/kg body weight; (**c**) of the control group.

**Figure 2:**
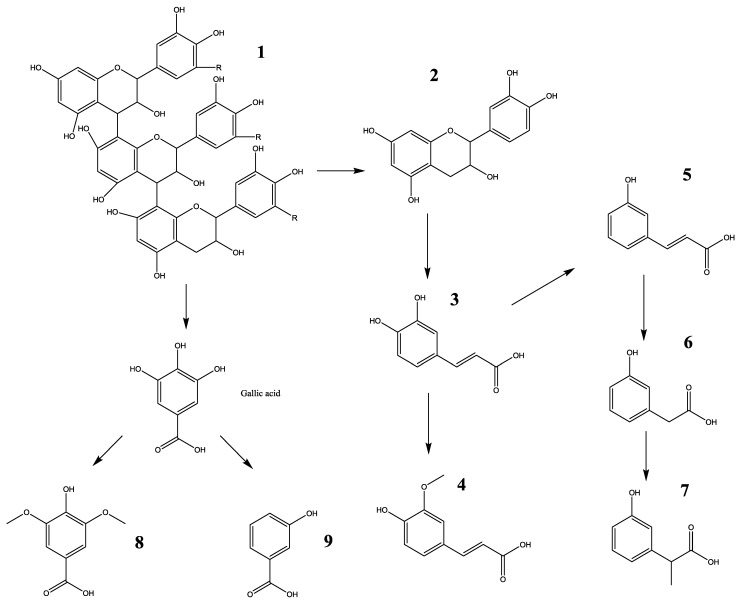
Proposed biotransformation pathway of polyphenol compounds in urine within 24 h, fed with LSOPC. **1**, LSOPC; **2**, catechin; **3**, caffeic acid; **4**, ferulic acid; **5**, *m*-coumaric acid; **6**, 3-hydroxyphenylacetic acid; **7**, 3-hydroxyphenylpropionic acid; **8**, syringic acid; **9**, 3-hydroxybenzoic acid.

Major metabolites of LSOPC in rat urine were analyzed after deconjugation by glucuronidase/sulfatase using HPLC-MS/MS and compared with (+)-catechin and phenolic acid standards (Figure 1, Table 1). The aromatic acids selected for analysis were those previously reported as microbial metabolites of procyanidins in *in vitro* and *in vivo* studies [20]. 3-Hydroxyphenylacetic acid, ferulic acid and *m*-coumaric acid were identified in both control and treatment urine samples (Table 1); while (+)-catechin, caffeic acid, syringic acid, 3-hydroxybenzoic acid and 3-hydroxyphenylpropionic acid were exclusively detected in the urine of rats orally administered with LSOPC (Table 1). However, the levels of all metabolites excreted in urine were very significantly increased by 300 mg/kg body weight of LSOPC (*p* < 0.01). 3-Hydroxyphenylacetic acid, 3-hydroxyphenylpropionic acid, ferulic acid and 3-hydroxybenzoic acid accounted for about 87% of the metabolites of LSOPC. The fragmentation pathway of this metabolic compound is shown in Figure 2.

It has been described that the radical-scavenging properties depend to the structures of polyphenols [21,22]. The inhibition effects of metabolites on AGE formation were tested in simulated physical models *in vitro*. The inhibition by LSOPC metabolites of fluorescence involved different mechanisms, such as the sugar source, metal chelation, and so on. It was reported that trapping reactive dicarbonyl and reactive oxygen species contributed to the inhibition of fluorescence [23].

**Table 1:** Metabolites excreted in urine within 24 h post-administration.

Compounds (nmol)	Retention time (min)	Parent ion (*m/z*)	Product ion (*m/z*)	Urinary excretion within 24 h post-administration	
				Control	300 mg/kg body weight
(+)-Catechin	12.7	289.5	244.8 (CO_2_ loss), 124.8 (HRF, 2 H_2_O loss)	ND ^c^	202.76 ± 20.33 *
Caffeic acid	14.8	179.3	134.7 (CO_2_ loss)	ND ^c^	22.46 ± 2.01 *
Syringic acid	15.2	197.2	152.9 (CO_2_ loss)	ND ^c^	113.64 ± 12.14 *
3-Hydroxybenzoic acid	15.7	136.9	92.9 (CO_2_ loss)	ND ^c^	695.65 ± 59.47 *
3-Hydroxyphenylacetic acid	16.1	151.3	106.9 (CO_2_ loss)	204.7	1046.05 ± 99.84 *
3-Hydroxyphenylpropionic acid	18.0	164.8	120.8 (CO_2_ loss)	ND ^c^	903.61 ± 89.05 *
Ferulic acid	20.5	192.8	148.8 (CO_2_ loss)	17.05	711.34 ± 70.86 *
*m*-Coumaric acid	22.2	163.1	118.8 (CO_2_ loss)	22.41	182.93 ± 16.58 *

Abbreviations: HRF, heterocyclic ring fission; QM, quinone methide cleavage. Values represent the concentrations of metabolites excreted within 24 h, and they were all expressed as the means ± SD (*n* *=* 5); ^c^ ND = not detected (limits of detector); * Indicates significant differences among two groups (*p* *<* 0.01).

### 3.3. Inhibition of AGE Formation

To investigate the effects of LSOPC metabolites on protein non-enzymatic glycation end product formation, the non-enzymatic glycation system *in vitro* was designed to monitor fluorescence at excitation and emission at wavelengths of 370 nm and 440 nm, respectively. As shown in Table 2, it was demonstrated that LSOPC and catechin had much better inhibition activity on fluorescent AGE formation than other metabolites. Aminoguanidine (AG) is a known AGE formation inhibitor. According to a previous study, the 1 mg/mL AG inhibition rate was only 57.2% ± 1.96% [8]. These results showed that LSOPC has a significant inhibitory activity on the formation of advanced glycation end products *in vitro* with respect to AG. Ferulic acid, caffeic acid and syringic acid possessed considerable inhibitory effects, as well.

**Table 2:** Anti-glycation activity and MGO scavenging activity of LSOPC and its metabolites.

Compounds	IC_50_ (mg/ mL)	MGO Scavenging
LSOPC	0.035 ± 0.004	81.24% ± 1.15%
(+)-Catechin	0.049 ± 0.019	78.25% ± 2.99%
Ferulic acid	0.741 ± 0.013	12.65% ± 0.53%
Caffeic acid	0.683 ± 0.012	14.80% ± 0.29%
Syringic acid	0.720 ± 0.017	20.61% ± 0.01%
*m*-Coumaric acid	4.150 ± 0.012	16.69% ± 0.37%
3-Hydroxybenzoic acid	2.318 ± 0.030	19.30% ± 0.13%
3-Hydroxyphenylacetic acid	1.126 ± 0.023	20.16% ± 0.25%
3-Hydroxyphenylpropionic acid	1.899 ± 0.014	20.72% ± 0.42%

IC_50_ concentrations ± SEM were calculated as the metabolite concentration required to reduce AGE formation by 50% as determined by regression analysis (*n* *=* 3).

### 3.4. Methylglyoxal Scavenging

**Figure 3:**
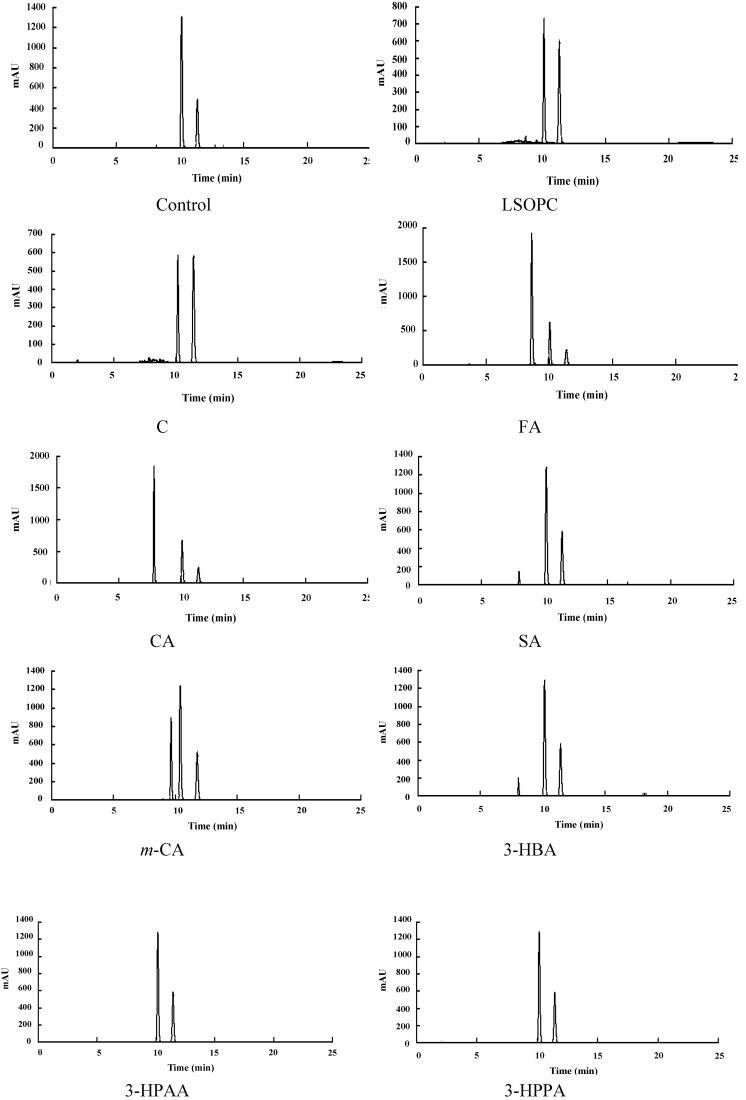
Chromatogram of the LSOPC-methylglyoxal adducts and metabolite-methylglyoxal adducts after incubation. C, (+)-catechin; FA, ferulic acid; CA, caffeic acid; SA, syringic acid; *m*-CA, *m*-coumaric acid; 3-HBA, 3-hydroxybenzoic acid; 3-HPAA, 3-hydroxyphenylacetic acid; 3-HPPA, 3-hydroxyphenylpropionic acid.

As to AGE inhibitors, their contributions to scavenging reactive carbonyls have been addressed in suppressing AGE formation under certain conditions *in vitro* [4,24]. In order to find out the inhibition mechanism of the metabolites, the MGO trapping abilities of LSOPC and its metabolites were investigated. The MGO decrease percentage (%) of each sample was shown in Table 2. The corresponding MGO trapping chromatograms were shown in Figure 3. The decreased amounts of MGO in LSOPC and catechin were 81.24% ± 1.15% and 78.25% ± 2.99%, respectively. In addition, LSOPC and catechin were found to be more reactive with MGO with respect to AG (positive control). The MGO trapping capacity of AG was 72.75% ± 1.47%, according to a previous study [8]; while other phenolic metabolites had weak MGO trapping activity, all below 21%, including ferulic acid, caffeic acid and syringic acid, which were supposed to have considerably high AGEs inhibitory effects. It was indicated that the capacity to scavenge reactive carbonyls plays an important role in the anti-glycation effects of LSOPC and catechin.

### 3.5. (DPPH) Radical Scavenging Activity and T-AOC of LSOPC and Its Metabolites

**Figure 4:**
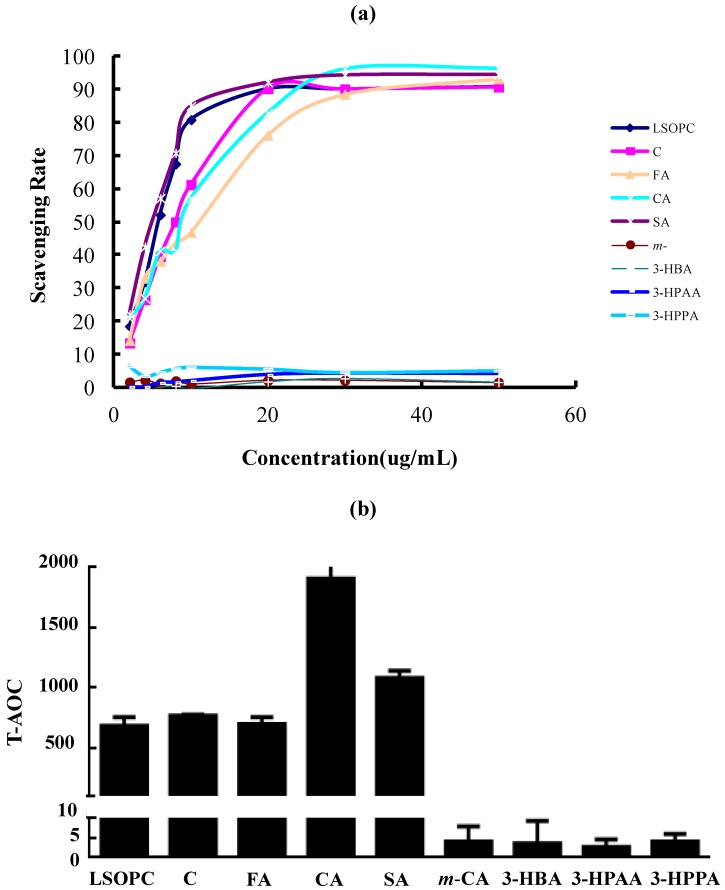
DPPH radical scavenging activity **(a)** and total antioxidant capability (T-AOC) **(b)** of LSOPC and its metabolites (*n* = 3). C, (+)-catechin; FA, ferulic acid; CA, caffeic acid; SA, syringic acid; *m*-CA, *m*-coumaric acid; 3-HBA, 3-hydroxybenzoic acid; 3-HPAA, 3-hydroxyphenylacetic acid; 3-HPPA, 3-hydroxyphenylpropionic acid.

Because free radical and oxidative reactions can accelerate glycation, the radical scavenging and antioxidant properties of LSOPC and its metabolites were considered. There was a dose-effect relation between the concentration of LSOPC, (+)-catechin, ferulic acid, caffeic acid, syringic acid and the scavenging rate on the DPPH radical (Figure 4a). The IC_50_ value of syringic acid was 4.817 ± 0.006 µg/mL, which held the strongest scavenging effect on DPPH among all of the metabolites. When LSOPC, (+)-catechin, ferulic acid and caffeic acid were at a concentration of 50 µg/mL, the scavenging rate were 90.85, 90.75, 93.26 and 96.47%, individually. The IC_50_ values of LSOPC, (+)-catechin, ferulic acid and caffeic acid were 5.483 ± 0.007, 7.927 ± 0.007, 10.742 ± 0.022 and 8.164 ± 0.014 µg/mL, respectively. Their scavenging abilities for DPPH radicals were as follows: ferulic acid < caffeic acid < (+)-catechin < LSOPC < syringic acid. However, *m*-coumaric acid, 3-hydroxybenzoic acid, 3-hydroxyphenylacetic acid and 3-hydroxyphenylpropionic acid almost have no scavenging effect on DPPH.

As we all know, the T-AOC of the metabolites corresponded to the activity of samples reducing Fe^3+^ to Fe^2+^, which were similar to the assay of ferric reducing antioxidant power (FRAP). The results were similar to those of DPPH scavenging activity. The results showed that the T-AOC of LSOPC, (+)-catechin, ferulic acid, caffeic acid and syringic acid were significantly higher than the others (Figure 4b). Among all of the metabolites, caffeic acid possessed the highest T-AOC activity.

This showed that the antioxidant activities of metabolites were positively correlated with their inhibitory capacity on AGE formation. Therefore, even though the MGO scavenging rate of ferulic acid, caffeic acid and syringic acid were low, they still had a relatively good inhibitory effect on AGE formation, probably because of their excellent antioxidant capacity.

## 4. Conclusions

It is well documented that the glycation of proteins contributes to the pathology of a number of chronic diseases, such as diabetes and Alzheimer dementia, and is also important in “normal” physiologic processes, such as aging [25,26,27]. A great deal of effort has been focused on identifying clinically useful inhibitors of protein AGEs to prevent glycation and to alleviate the phenotype of these diseases. In this study, eight metabolites of LSOPC were identified by HPLC-MS/MS. These metabolites played an important role in the health effects of LSOPC, which was rapidly metabolized by intestinal flora. Thus, more attention should be paid to investigate the inhibitory ability of metabolites on AGE formation in a simulated physical environment. It was demonstrated that (+)-catechin, ferulic acid, caffeic acid and syringic acid were able to inhibit AGE formation efficiently. Among them, (+)-catechin has the highest inhibitory effect, close to LSOPC. It is supposed that catechin is the major metabolite of lotus seedpod oligomeric procyanidins, providing a significant inhibitory effect on the formation of AGEs *in vivo*, or it may work with other metabolites to achieve a great inhibitory effect. It also was shown that the antioxidant activity of metabolites contributed to their AGE inhibition capacities. These effects likely contribute to the beneficial health effects associated with lotus seedpod utilization.
